# End-point rapid detection of total and pathogenic *Vibrio parahaemolyticus* (*tdh*^+^ and/or *trh1*^+^ and/or *trh2*^+^) in raw seafood using a colorimetric loop-mediated isothermal amplification-xylenol orange technique

**DOI:** 10.7717/peerj.16422

**Published:** 2024-01-03

**Authors:** Aekarin Lamalee, Soithong Saiyudthong, Chartchai Changsen, Wansika Kiatpathomchai, Jitra Limthongkul, Chanita Naparswad, Charanyarut Sukphattanaudomchoke, Jarinya Chaopreecha, Saengchan Senapin, Wansadaj Jaroenram, Sureemas Buates

**Affiliations:** 1 Department of Microbiology, Faculty of Science, Mahidol University, Bangkok, Thailand; 2 Institute of Food Research and Product Development, Kasetsart University, Bangkok, Thailand; 3 Bioengineering and Sensing Technology Research Team, National Center for Genetic Engineering and Biotechnology (BIOTEC), National Science and Technology Development Agency (NSTDA), Pathum Thani, Thailand; 4 Center of Excellence for Shrimp Molecular Biology and Biotechnology, Faculty of Science, Mahidol University, Bangkok, Thailand; 5 National Center for Genetic Engineering and Biotechnology (BIOTEC), National Science and Technology Development Agency (NSTDA), Pathum Thani, Thailand

**Keywords:** Loop-mediated isothermal amplification-xylenol orange (LAMP-XO), *V. parahaemolyticus*, Seafood, Rapid detection, Colorimetric technique

## Abstract

**Background:**

*Vibrio parahaemolyticus* is the leading cause of bacterial seafood-borne gastroenteritis in humans worldwide. To ensure seafood safety and to minimize the occurrence of seafood-borne diseases, early detection of total *V. parahaemolyticus* (pathogenic and non-pathogenic strains) and pathogenic *V. parahaemolyticus* (*tdh*^+^ and/or *trh1*^+^ and/or *trh2*^+^) is required. This study further improved a loop-mediated isothermal amplification (LAMP) assay using xylenol orange (XO), a pH sensitive dye, to transform conventional LAMP into a one-step colorimetric assay giving visible results to the naked eye. LAMP-XO targeted *rpoD* for species specificity and *tdh, trh1*, and *trh2* for pathogenic strains. Multiple hybrid inner primers (MHP) of LAMP primers for *rpoD* detection to complement the main primer set previously reported were designed by our group to maximize sensitivity and speed.

**Methods:**

Following the standard LAMP protocol, LAMP reaction temperature for *rpoD*, *tdh*, *trh1*, and *trh2* detection was first determined using a turbidimeter. The acquired optimal temperature was subjected to optimize six parameters including dNTP mix, betaine, MgSO_4_, *Bst* 2.0 WarmStart DNA polymerase, reaction time and XO dye. The last parameter was done using a heat block. The color change of the LAMP-XO result from purple (negative) to yellow (positive) was monitored visually. The detection limits (DLs) of LAMP-XO using a 10-fold serial dilution of gDNA and spiked seafood samples were determined and compared with standard LAMP, PCR, and quantitative PCR (qPCR) assays. Subsequently, the LAMP-XO assay was validated with 102 raw seafood samples and the results were compared with PCR and qPCR assays.

**Results:**

Under optimal conditions (65 °C for 75 min), *rpoD*-LAMP-XO and *tdh*-LAMP-XO showed detection sensitivity at 10^2^ copies of gDNA/reaction, or 10 folds greater than *trh1*-LAMP-XO and *trh2*-LAMP-XO. This level of sensitivity was similar to that of standard LAMP, comparable to that of the gold standard qPCR, and 10-100 times higher than that of PCR. In spiked samples, *rpoD*-LAMP-XO, *tdh*-LAMP-XO, and *trh2*-LAMP-XO could detect *V. parahaemolyticus* at 1 CFU/2.5 g spiked shrimp. Of 102 seafood samples, LAMP-XO was significantly more sensitive than PCR (*P* < 0.05) for *tdh* and *trh2* detection and not significantly different from qPCR for all genes determined. The reliability of *tdh*-LAMP-XO and *trh2*-LAMP-XO to detect pathogenic *V. parahaemolyticus* was at 94.4% and 100%, respectively.

**Conclusions:**

To detect total and pathogenic *V. parahaemolyticus*, at least *rpoD*-LAMP-XO and *trh2*-LAMP-XO should be used, as both showed 100% sensitivity, specificity, and accuracy. With short turnaround time, ease, and reliability, LAMP-XO serves as a better alternative to PCR and qPCR for routine detection of *V. parahaemolyticus* in seafood. The concept of using a one-step LAMP-XO and MHP-LAMP to enhance efficiency of diagnostic performance of LAMP-based assays can be generally applied for detecting any gene of interest.

## Introduction

Seafood products are widely consumed globally and play an important role in the economic market. Even though seafood consumption is suggested as a part of a healthy diet (Hosomi, Yoshida & Fukunaga, 2012), several pathogens related with adverse human health effects including gastrointestinal diseases are present in seafood (Choudhury et al., 2022). Because of the growth in global consumption of seafood products, inspection of seafood quality is the primary way to inhibit contamination from seafood-borne pathogens (FAO, 2020; Choudhury et al., 2022). Among seafood-borne pathogens, *Vibrio parahaemolyticus*, a Gram-negative, rod-shaped bacterium, is a leading cause of seafood-borne bacterial gastroenteritis in humans. Global gastroenteritis caused by *V. parahaemolyticus* is normally due to consumption of raw or undercooked contaminated seafood, especially shellfish (Raszl et al., 2016; Centers for Disease Control and Prevention , 2019). *Vibrio parahaemolyticus* is naturally present in marine environments globally. It is often associated with aquatic products as well as shellfish, shrimp, and fish (Letchumanan et al., 2019; Changsen et al., 2023). This bacterium accounts for a considerable increase of seafood-borne infections worldwide (Letchumanan, Chan & Lee, 2014).

*Vibrio parahaemolyticus* possesses different virulence factors. The major ones are thermostable direct hemolysin (TDH) encoded by the *tdh* gene and TDH-related hemolysins (TRH) encoded by the *trh* gene, both of which are present mostly in clinical strains (Raghunath, 2015; Cai & Zhang, 2018). TDH is a heat-resistant, pore-forming toxin comprising of 156 amino acids (Li et al., 2019). It forms pores on erythrocyte membrane, allowing water and ions to flow through the membrane leading to erythrocyte lysis. It also exerts several mainly biological activities including cytotoxicity, cardiotoxicity, and enterotoxicity (Cai & Zhang, 2018). TRH is a heat-labile toxin composing of 189 amino acids. It has similar biological activities to the TDH (Honda, Ni & Miwatani, 1988; Nishibuchi et al., 1989). The *trh* and *tdh* genes share 54.8–68.8% identity in their sequences (Kishishita et al., 1992). The *trh* gene can be subdivided into *trh1* and *trh2* which share 84% homology (Nishibuchi et al., 1989; Kishishita et al., 1992). It is known that all pathogenic *V. parahaemolyticus* strains harbor *tdh* and/or *trh* genes while the non-pathogenic strains lack both *tdh* and *trh* genes (Honda & Iida, 1993; Nishibuchi & Kaper, 1995). Therefore, *tdh* and *trh* genes are considered as molecular markers for *V. parahaemolyticus* pathogenicity.

Many *V. parahaemolyticus* infections are epidemiologically related with seafood consumption, especially shellfish contaminated with pathogenic strains (Raszl et al., 2016). Accordingly, controlling *V. parahaemolyticus* contamination in seafood can effectively prevent seafood-borne diseases. Moreover, ecological and epidemiological surveillance on the prevalence of pathogenic *V. parahaemolyticus* strains is needed since virulence genes can be horizontally transferred to non-virulent strains (Waldor & Mekalanos, 1996). Several methods are available for detection of *tdh* and *trh* genes of *V. parahaemolyticus* including polymerase chain reaction (PCR), quantitative PCR (qPCR), and droplet digital PCR. However, these advanced methods are time-consuming and need expensive apparatus, expensive reagents as well as trained personnel (Xu et al., 2018; Lei et al., 2020; Guan et al., 2021). Therefore, developing a simple diagnostic method with high sensitivity and specificity would be essential.

Loop-mediated isothermal amplification (LAMP), a DNA-based amplification assay that amplifies nucleic acids under a single temperature is one such tool, having the potential to be a DNA-based point-of-care (POC) diagnostic method (Notomi et al., 2000). Unlike PCR and qPCR, LAMP assay can be done in a heat block or water bath. Recently, many LAMP assays have been employed to detect *tdh* and *trh* genes of *V. parahaemolyticus* (Yingkajorn et al., 2014; Yan et al., 2017; Anupama et al., 2021). In general, the detection of LAMP products/amplicons can be done *via* (1) a direct observation of the white precipitate (turbidity) of magnesium pyrophosphate, Mg_2_P_2_O_7_, (a by-product of LAMP reaction) and (2) indirect methods including, turbidity measurement by a turbidimeter (EIKEN Co. Ltd.), fluorescent observation under UV light, agarose gel electrophoresis (AGE), and lateral flow dipstick (LFD) detection (Mori et al., 2001; Prompamorn et al., 2011; Wong et al., 2018). However, the direct method monitoring with the naked eye can give ambiguous readout, especially in weakly positive samples. Although this weakness can be avoided by using a turbidimeter or a UV light transilluminator, such devices are costly and required additional steps to work on (Wong et al., 2018). Likewise, AGE and LFD require additional costs, post-amplification steps and opening of a reaction tube, thereby risking possible cross-contamination. To overcome these drawbacks and to improve the overall diagnostic performance of direct LAMP assay readout, a simpler colorimetric method was explored. In 2019, Jaroenram, Cecere & Pompa (2019) exploited xylenol orange (XO) to detect *Escherichia coli* DNA. The XO is a low-cost pH indicator whose color changes from violet to yellow at pH < 6.7, allowing a chance to detect the progress of LAMP reaction directly *via* the naked eye. To illustrate, during LAMP amplification, large amounts of Mg_2_P_2_O_7_ and protons (H^+^) are generated, resulting in a significant pH drop from initial alkaline pH values (8.5–9.0) to a final acidic pH value of approximately 6.0–6.5 when LAMP reaction is performed in low concentrations of buffer or non-buffered solution (Tanner, Zhang & Evans Jr, 2015). In the presence of XO, the presence of target DNA in test samples will trigger the change of the reaction hue from purple to yellow (positive readout/LAMP amplicon buildup). A lack of detection targets will present the original violet hue of the reaction (negative result). The result can be seen easily by the naked eye. To the best of our knowledge, LAMP-XO strategy has not been applied to detect *Vibrio* spp. Herein, we have developed LAMP-XO assay, and validated whether it would rapidly, sensitively, and accurately detect total *V. parahaemolyticus* (pathogenic and non-pathogenic strains) and pathogenic *V. parahaemolyticus* (*tdh*^+^ and/or *trh1*^+^ and/or *trh2*^+^) in raw seafood. Based on 102 raw seafood samples, the assay is as efficient as the existing molecular assays but cheaper, faster, and easier to use, thus more suitable for point of care (POC) testing.

## Materials & Methods

### Bacterial strains, culture conditions, and genomic DNA template preparation

*Vibrio parahaemolyticus* reference stains, DMST 15285 (*tdh*^+^/*trh*^−^) (obtained from the Department of Medical Science, Ministry of Public Health, Nonthaburi Province, Thailand), TISTR 1596 or ATCC 17802 (*tdh*^−^/*trh2*^+^) (obtained from the Thailand Institute of Scientific and Technological Research), and Vp10/5 (*tdh*^+^/*trh1*^+^) (obtained from an oyster, Chatuchak District, Bangkok, Thailand in 2018) were utilized as the positive controls to optimize and validate LAMP-XO assay. The reference stains were cultured in 5 mL tryptic soy broth (TSB) (Merch KGaA, Darmstadt, Germany) supplemented with 2% NaCl (BDH Poole, USA) in a shaking incubator at 250 rpm for 16 h at 37 °C. A volume of three mL of the culture broth was transferred into a 1.5-mL microcentrifuge tube and centrifuged at 10,000 rpm for 2 min to remove the supernatant. The pellet was resuspended in Tris-EDTA (TE) buffer (Invitrogen, Grand Island, NY, USA), and subjected to genomic DNA (gDNA) extraction using GenUP™ kit (Biotechrabbit, Berlin, Germany) according to the manufacturer’s protocol. The gDNA concentration was measured using a nanodrop (DS-11 FX+, spectrophotometer/fluorometer; DeNovix, Wilmington, DE, USA). The gDNA solution was aliquoted and kept at −80 °C.

### Sample collection, *V. parahaemolyticus* detection, storage, recovery, and gDNA template preparation

A total of 102 raw seafood samples were used in this study. Of the 102 samples, 83 were purchased from fresh markets and supermarkets in Bangkok, Thailand of which 30 and 53 were purchased in 2018 and 2021 to 2022, respectively. Of the 102 samples, 16 samples were *V. parahaemolyticus* isolates obtained from Pacific white shrimp collected from different shrimp farms in eastern Thailand in 2013 and three samples were *V. parahaemolyticus* isolates obtained from Pacific white shrimp collected from North Vietnam in 2016. Eighty three samples purchased from markets comprised of six crabs (five blue swimming crabs and one red swimming crab), 15 fish (five groupers, five giant sea perches, two mackerels, one ornate threadfin bream, one bluefin tuna, and one salmon), 31 mollusc shellfish (15 oysters, six green mussels, five blood cockles, two short-necked clams, two spiral babylon snails, and one scallop), 21 shrimp (one giant tiger prawn and 20 Pacific white shrimp) and 10 squids (six splendid squids, three octopuses, and one giant squid tentacle). For *V. parahaemolyticus* detection of the purchased seafood, the samples were separately put into a sterile plastic bag and taken to a laboratory in a cooler bag containing ice and were handled within 2 h after sample purchasing. Briefly, 2.5 g of each sample was cut aseptically and immersed into 22.5 mL of TSB (Merch KGaA, Germany) supplemented with 2% NaCl (BDH Poole, Rahway, NJ, USA) for bacterial enrichment. The sample was gently mixed by hand and incubated at room temperature (RT) for 30 min before being removed from culture broth which was further incubated at 37 °C for 16 h. A total of three mL of culture broth was subjected to gDNA isolation. The extracted gDNA was used as a template for LAMP-XO, standard LAMP, qPCR, and conventional PCR assays. For *V. parahaemolyticus* isolation, one loop full of the culture broth was streaked onto thiosulfate-citrate-bile salts-sucrose agar (TCBSA) (BD, Sparks, USA) on which *V. parahaemolyticus* produced opaque and blue-green color with 2–3 mm in diameter colonies. The presumptive *V. parahaemolyticus* colonies were picked up separately and transferred to CHROMagar™ Vibrio (CHROMagar, Paris, France) on which *V. parahaemolyticus* produced mauve color colonies. For long-term storage, a single mauve colony of *V. parahaemolyticus* on CHROMagat™ Vibrio was cultured in TSB (Merch KGaA, Germany) supplemented with 2% (w/v) NaCl (BDH Poole, Rahway, NJ, USA) at 37 °C for overnight. A total of 400 µL of 50% glycerol (Sigma-Aldrich, St.Louis, MO, USA) and 600 µL of an overnight culture were mixed aseptically in a sterile 1.5-mL microcentrifuge tube and kept at −80 °C. For bacterial recovery, ice crystals on top of a glycerol stock were aseptically scraped and transferred into TSB supplemented with 2% NaCl and incubated in a shaker incubator at 37 °C with 250 rpm shaking for overnight. The presumptive culture was subjected to species-specific *rpoD*-qPCR assay for confirmation of species.

### Optimization and validation of LAMP-xylenol orange assay

Optimization of LAMP-xylenol orange (LAMP-XO) assay was carried out using 4 sets of previously described primers targeting four different genes: Set 1 for *rpoD* gene for total *V. parahaemolyticus* (pathogenic and non-pathogenic strains) detection (Nemoto et al., 2011; Lamalee et al., 2023) and sets 2–4 for *tdh, trh1*, and *trh2* genes, respectively, for *V. parahaemolyticus* pathogenic strain detection (Table 1) (Nemoto et al., 2009; Yamazaki et al., 2010). The optimization for each gene was done separately. For *rpoD* detection, in addition to the main primers utilized, we designed four additional primers (loop forward and loop backward 2; F1c2 and B1c2, and forward inner and backward inner 2; FIP2 and BIP2) (Table 1) to further improve the reaction kinetics (Jaroenram et al., 2022). The primers were examined for possible cross dimerization by basic local alignment search tool (BLAST) (https://blast.ncbi.nlm.nih.gov/Blast.cgi).

**Table 1:** LAMP primers and conditions used in this study.

**Primer name**	**Sequence (5′ to 3′)**	**Target gene**	**Concentration** ** (** **pmol/µL** **)**	**Reference**
*rpoD*-FIP	TGAATACGTCTAGCATCATTTCGTCGATCAATGAGTACGGCTACGA	*rpoD*	20	Nemoto et al. (2011)
*rpoD*-BIP	ACAGCAATGGATCGCGTTCCGATTTCTTCGGCATTTTGCC		20	
*rpoD*-F3	ACCAGCTACGCAGCACA		20	
*rpoD*-B3	CACTTGATTCGTTACCAGTGAATAGG		20	
*rpoD-* LF	GCAACGGTTGCTTTCGG		20	
*rpoD-* LB	GTTTGATCATGAAGTCTGTGG		20	
Extra primer				Lamalee et al. (2023)
*rpoD*-FIB2	TGGTGTTAGACGGAATTCTTTTCGATCAATGAGTACGGCTACGA		20	
*rpoD*-BIP2	CACCTAGTGAACGAACTTCTTTTCGATTTCTTCCCCATTTTGCC		20	
*rpoD*-F1c2	TGGTGTTAGACGGAATTC		20	
*rpoD*-B1c2	CACCTAGTGAACGAACTTC		20	
*tdh*-FIP	CTTATAGCCAGACACCGCTGCGGTTGACATCCTACATGACTGTG	*tdh*	40	Nemoto et al. (2009)
*tdh*-BIP	CGGTCATTCTGCTGTGTTCGTTCTTCACCAACAAAGTTAGCTACAG		40	
*tdh*-F3	GTCTCTGACTTTTGGACAAACCG		10	
*tdh*-B3	CTACATTAACAAAATATTCTGGAGTTTCATCC		10	
*tdh*-LF	CCGCTGCCATTGTATAGTCTTT		40	
*tdh*-LB	CAGATCAAGTACAACTTCAACATTCCT		40	
*trh1*-FIP	AGGCTTGTTTTTTCTGATTTTGTGACTACACAATGGCTGCTCT	*trh1*	40	Yamazaki et al. (2010)
*trh1*-BIP	TCTTCTGTTAGTGATTTCGTTGGTTTTCATCCAAATACGTTACACT		40	
*trh1*-F3	GCGCCTATATGACGGTAA		5	
*trh1*-B3	ACATTGACGAAATATTCTGGC		5	
*trh1*-LF	AGACCGTTGARAGGCC		20	
*trh2-* FIP	CCGATTGACCGTATACATCTTTGTTGTGGAGGACTATTGGACAA	*trh2*	40	Yamazaki et al. (2010)
*trh2-* BIP	TCAAAGTGGTTAAGCGCCTATATGCCATSTTTATAACCAGAAAGAGC		40	
*trh2-* F3	CATCAATACCTTTTCCTTCTCC		5	
*trh2-* B3	GCTTGTTTTCTCTGATTTTGTG		5	
*trh2-* LF	TGGTTTTCTTTTTATGKTTCGGT		20	
*trh2-* LB	ATGGTCAYAACTATACRATGGC		20	

Briefly, the protocol (Table S1) was done in a 25-µL reaction mixture containing each target-specific primer set (forward inner primer (FIP), backward inner primer (BIP), forward outer primer (F3), backward outer primer B3), forward loop primer (LF), and backward loop primer (LB)) at different amounts (Table 1), dNTP mix (New England Biolabs, Ipswich, MA, USA), betaine (Sigma-Aldrich, St.Louis, MO, USA) MgSO_4_ (New England Biolabs, Ipswich, MA, USA), *Bst* 2.0 WarmStart DNA polymerase (New England Biolabs, Ipswich, MA, USA), 2.5 µL of 1× low-buffer solution with pH 8.5 (100 mM (NH_4_)_2_SO_4_, 500 mM KCl, 20 mM MgSO_4_, and 1% Tween-20), and 1 µL of a gDNA template. The final volume was adjusted to 25 µL using UltraPure™ distilled water (DW) (Invitrogen, Grand Island, Germany). The negative control containing only DW (no gDNA templates) was included in each run. LAMP reaction was done in a Loopamp Realtime Turbidimeter LA-320C (Eiken Chemical Co Ltd, Tokyo, Japan) at a given condition (temperature and time) followed by DNA polymerase inactivation at 80 °C for 5 min. Following this initial protocol, the optimal incubation temperature was first determined, in that the LAMP reactions were carried out at various temperatures (60, 63, and 65 °C) for 75 min. The obtained optimal temperature was then subjected to optimizing six respective parameters: dNTP mix (1.2–1.8 mM), betaine (0.2–0.8 M), MgSO_4_ (4–10 mM), *Bst* 2.0 WarmStart DNA polymerase (6–12 U), reaction time (30, 45, 60, and 75 min), and XO dye, (0.03–0.12 mM) (Table S2). The last parameter was performed in a heat block, and the result was inspected visually. Color of LAMP-XO was changed from purple to yellow in a positive test while color was still purple in a negative test. To confirm LAMP-XO results, LAMP amplicons were analyzed by 3% agarose gel electrophoresis (AGE) (Vivantis, Malaysia), stained with ethidium bromide (Invitrogen, Waltham, MA, USA) and visualized under UV illumination. Each parameter used 1 µL gDNA of 10^6^ copies/µL/reaction as a template except for the incubation temperature and time that used 1 µL aliquot of 10-fold serially diluted DNA (10^4^, 10^3^, 10^2^, 10 copies/µL) instead. The DNA copy has been calculated using the formular “amount of DNA (ng) × 6.022 × 10^23^/ length of a DNA template (bp) × 1  × 10^9^ × 650” (https://www.technologynetworks.com/tn/tools/copynumbercalculator). For each primer set/target gene, any given temperature, time, and components’ concentration that maximize DNA amplification based on signal intensities by the turbidimeter and the degree of color change from purple (negative) to yellow (postitive) was selected to establish the standard LAMP-XO protocol.

### Standard LAMP assay

The standard LAMP assay was non-colorimetric, adopted from the standard LAMP protocol suggested by New England Company Ltd. (https://international.neb.com/protocols/2014/06/17/loop-mediated-isothermal-amplification-lamp). It was used as a control assay to test the efficiency of LAMP-XO. Its reaction components were almost similar to those of the optimized LAMP-XO, only excluding XO, and the low-buffer that was substituted by 1× ThermoPol-supplied reaction buffer (20 mM Tris-HCl, 10 mM (NH_4_)_2_SO_4_, 10 mM KCl, 2 mM MgSO_4_ and 0.1% Triton X-100, pH 8.8). The LAMP reaction was performed in a Loopamp Realtime Turbidimeter LA-320C (Eiken Chemical Co Ltd, Tokyo, Japan), at 65 °C (75 min for *rpoD*, *tdh*, *trh1*, and *trh2*). The results were reported in a real-time amplification plot format. For result confirmation, LAMP products were inspected using 3% AGE.

### Conventional PCR assay

Conventional PCR assay was performed using species-specific *toxR* primers for *V. parahaemolyticus* detection and *tdh, trh1*, and *trh2* primers for *V. parahaemolyticus* pathogenic strain detection (Table S3) (Tada et al., 1992; Kim et al., 1999; Messelhäusser et al., 2010). PCR reaction was carried out in a 25-µL reaction mixture containing 12.5 µL GoTaq^®^ Green Master Mix solution (Promega, Madison, USA), 0.4 µM each forward primer and backward primer for *toxR*, *tdh, trh1*, and *trh2* primers and 1 µL of a gDNA template. A final volume of a 25-µL reaction mixture was adjusted with UltraPure™ DW (Invitrogen, Grand Island, Germany). The PCR amplification was done in T100 Thermal Cycler No. 186-1096 (Bio-Rad, Hercules, CA). PCR products were analyzed using 1.5% AGE (Vivantis, Malaysia), stained with ethidium bromide (Sigma-Aldrich, St.Louis, MO, USA) and observed under a UV light. A positive control and a negative control were included in each run.

### Quantitative PCR assay

A quantitative PCR (q-PCR) assay was done using species-specific *rpoD* primers for *V. parahaemolyticus* detection and *tdh*, *trh1*, and *trh2* primers for *V. parahaemolyticus* pathogenic strain detection (Table S4) (Nemoto et al., 2009; Messelhäusser et al., 2010; Yamazaki et al., 2010; Nemoto et al., 2011). A 20-µL reaction mixture consisted of 10 µL of 2× KAPA SYBR FAST qPCR Master Mix Universal (Sigma-Aldrich, St.Louis, MO, USA), 10 mM each forward primer and backward primer, and 1 µL aliquot of 20 ng/µL of a gDNA template. A final volume was adjusted using UltraPure™ DW (Invitrogen, Grand Island, Germany). The qPCR amplification was carried out in a Rotor-Gene Q R10116106 (Qiagen Hilden, Germany). A positive control, a negative control, and a standard curve were included in each run.

### Comparative detection limit of LAMP-XO, standard LAMP, conventional PCR, and qPCR assays by gDNA

The concentrations of gDNA from the reference strains, DMST 15285, TISTR 1596 or ATCC 17802, and Vp10/5, were measured using a nanodrop (DS-11 FX+, spectrophotometer/fluorometer; DeNovix, Wilmington, DE, USA) and subjected to serial dilution in a 10-fold manner from 10^4^–10^0^ copies/µL. The DNA solution was used as a template for LAMP-XO, standard LAMP, qPCR, and conventional PCR assays.

### Comparative detection limit of LAMP-XO, standard LAMP, conventional PCR, and qPCR assays by spiked seafood samples

Three Pacific white shrimp from a supermarket were sterilized by autoclaving. Lack of *V. parahaemolyticus* contamination was later confirmed by a culture method using TCBSA (Difco Laboratories, Sparks, MD, USA). Briefly, 2.5 g of each Pacific white shrimp was spiked with one mL aliquot of a 10-fold serial dilution of overnight culture of *V. parahaemolyticus* reference strains to generate inoculating levels of 10^4^–10^0^ colony-forming unit (CFU)/2.5 g sample. The inoculating levels were calculated based on the assumption of OD_600_ = 1 = 8  × 10^8^ CFU/mL. A negative control was a non-spiked sample. Each sample was adjusted to 22.5 mL using TSB supplemented with 2% NaCl, mixed gently by hands and stored at RT for 30 min. All samples were further incubated at 37 °C for 4 h prior to gDNA extraction. The obtained gDNA was subjected to LAMP-XO, qPCR, and conventional PCR assays.

### Detection of total *V. parahaemolyticus***(pathogenic and non-pathogenic strains) and pathogenic***V. parahaemolyticus* (*tdh*^+^**and/or***trh1*^+^**and/or***trh2*^+^) **in raw seafood samples by LAMP-XO andstatistical analysis**

For LAMP-XO validation using seafood samples, LAMP-XO reaction for each target gene was carried out using optimal reagent concentrations and conditions as shown in Table 2. Final results of LAMP-XO validation were cross-compared with conventional PCR and qPCR. Specimens were classified as true positive, true negative, false positive, or false negative for each test under evaluation compared to qPCR as a gold standard. The clinical sensitivity, specificity, positive predictive values (PPV), negative predictive values (NPV), diagnostic accuracy, percent overall agreement (POA), and 95% CIs of LAMP-XO, conventional PCR, and qPCR assays were analyzed by SAS software (SAS Institute Inc., Cary, NC, USA). The clinical sensitivity was calculated as (number of true positives)/(number of true positives + number of false negatives) × 100, and the clinical specificity was calculated as (number of true negatives)/(number of true negatives + number of false positives) × 100. The PPV was calculated as (number of true positives)/(number of true positives + number of false positives) × 100, and the NPV was calculated as (number of true negatives)/(number of true negatives + number of false negatives) × 100. The accuracy was calculated as (number of true positives + number of true negatives)/(total number of patients) × 100. The degree of agreement between two diagnostic tests was measured by the concordance response rate (percentage of responses with both positive or both negative results). The POA indicates the percentage of relationship between varied results of comparative and reference methods. Note that the clinical (statistical) sensitivity refers to the ability of the validated assay to correctly identify real positive samples (*V. Parahaemolyticus* contaminated samples), while the clinical (statistical) specificity refers to the ability of the validated assay to correctly identify real negative samples (*V. Parahaemolyticus*-free samples). The significant difference between two detection methods, *i.e.,* LAMP-XO and qPCR and LAMP-XO and conventional PCR for each gene was analyzed with a McNemar chi-square test and a *P* value < 0.05 was considered significant.

**Table 2:** LAMP-XO conditions, primers, and reagents’ concentrations.

**Target gene**	**Optimal parameter of LAMP-XO reaction in a final volume of 25 µL**							
	**Temperature** **(° C)**	**dNTP mix** **(mM)**	**MgSO**_**4**_ **(mM)**	**Betaine** **(M)**	***Bst*** ** 2.0 WS DNA polymerase (U)**	**Reaction time (min)**	**XO** **(mM)**	**Low buffer**
*rpoD*	65	1.6	8	0.8	8	75	0.06	1 ×
*tdh*	65	1.4	8	0.8	8	75	0.06	1 ×
*trh1*	65	1.2	8	0.8	8	75	0.03	1 ×
*trh2*	65	1.2	8	0.8	8	75	0.03	1 ×

**Notes.**

The concentrations of primers for each target gene are shown in Table 1.

## Results

### Optimization of LAMP-XO assay conditions

LAMP reactions for each target gene (*rpoD*, *tdh*, *trh1*, and *trh2*) (Table 1) were performed in a low-buffer solution (pH 8.5) based on the method previously reported to leverage the effect of a pH drop for colorimetric product (Jaroenram, Cecere & Pompa, 2019). Since LAMP reaction standard temperatures range from 60–65 °C, the incubation temperature at which a template could be best amplified was firstly optimized, followed by LAMP reagents, reaction time, and XO concentrations (Table S2).

In the LAMP reaction cocktail, the optimal XO concentration would qualitatively generate the most substantial colorimetric change from purple (negative) to yellow (positive) upon a decrease in pH (< 6.7) in the presence of the LAMP reaction by-products. The XO concentration producing the most distinctive color difference between the positive and negative test results was selected (Table S5). Using a high XO concentration is a concern since it interferes in a weak positive reaction. Overall, different target genes have different components’ concentrations, except the incubation temperature (65 °C) and amplification time (75 min) which is in common as reported in Table 2. The performance of optimal LAMP-XO was compared with conventional PCR and qPCR assays (Table S6).

### Comparative detection limit of LAMP-XO, standard LAMP, conventional PCR, and qPCR assays using a 10-fold serial dilution of gDNA

**Figure 1:**
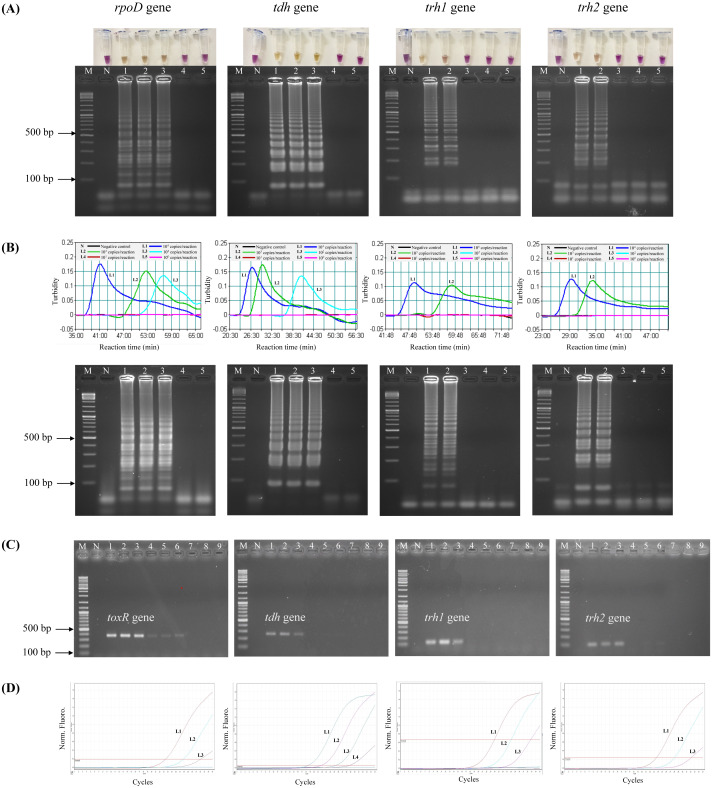
Comparative sensitivity of LAMP-XO, standard LAMP, conventional PCR, and qPCR assays for the detection of total *V. parahaemolyticus* and pathogenic *V. parahaemolyticus* using 10^4^–10^0^ copies of gDNA/reaction. The *rpoD* primer was used for total *V. parahaemolyticus* detection in LAMP-XO, standard LAMP, and qPCR assays while the *toxR* primer was used in PCR assay. The *tdh*, *trh1*, and *trh2* primers were used for pathogenic *V. parahaemolyticus* detection in all assays. (A) LAMP-XO assay detected by the naked eye (top) and AGE (bottom); (B) standard LAMP assay detected by a turbidimeter (top) and AGE (bottom); (C) conventional PCR assay detected by AGE; (D) Quantitative PCR (q-PCR) assay. (A), (B), and (D) L1: 10^4^ copies/reaction, L2: 10^3^ copies/reaction, L3: 10^2^ copies/reaction, L4: 10^1^ copies/reaction, L5: 10^0^ copies/reaction; (C) L1-L3: 10^4^ copies/reaction, L4-L6: 10^3^ copies/reaction, L7-L9: 10^2^ copies/ reaction; M: 1 kb DNA ladder, N: Negative control.

To test the analytical sensitivity of the assays, LAMP-XO, standard LAMP, PCR, and qPCR assays were conducted for each target gene/primer set with the same set of a gDNA template ranging from 10^4^–10^0^ copies/reaction (Table 3, Fig. 1). Primers targeting *rpoD* were used for total *V. parahaemolyticus* (pathogenic and non-pathogenic strains) detection in LAMP-XO, standard LAMP, and qPCR assays while primers targeting *toxR* were used for total *V. parahaemolyticus* (pathogenic and non-pathogenic strains) detection in PCR. Primers targeting *tdh*, *trh1*, and *trh2* were used for pathogenic *V. parahaemolyticus* (*tdh*^+^ and/or *trh1*^+^ and/or *trh2*^+^) detection. For LAMP-XO assay, *rpoD* and *tdh* primers indicated positive amplification at 10^4^–10^2^ copies, while *trh1* and *trh2* primers did at 10^4^–10^3^ copies. Thus, the DLs of the naked eye detection of LAMP-XO for *rpoD* and *tdh* genes were 10^2^copies/reaction and for *trh1* and *trh2* genes were 10^3^ copies/reaction (Fig. 1A, top). These colorimetric results were in agreement with those confirmed by AGE (Fig. 1A, bottom). Standard LAMP assay determined DLs of 10^2^ copies/reaction for *rpoD* and *tdh* and 10^3^ copies/reaction for *trh1* and *trh2* for both turbidity measurement (Fig. 1B, top) and AGE (Fig. 1B, bottom). PCR produced DLs at 10^3^ copies for *toxR* and 10^4^ copies for *tdh*, *trh1*, and *trh2* (Fig. 1C), while qPCR assay produced DLs at 10^1^ copies/reaction for *tdh* and 10^2^ copies/reaction for *rpoD*, *trh1*, and *trh2* (Fig. 1D).

**Table 3:** Comparative detection limit (DL) of LAMP-XO, standard LAMP, conventional PCR, and qPCR assays using a 10-fold serial dilution of gDNA (10^4^–10^0^ copies/reaction).

**Primer**	**10-fold serial dilution of gDNA (10** ^**4**^ **-** **10** ^**0**^ ** copies/reaction)**			
	**LAMP-XO**	**Standard LAMP**	**Conventional PCR**	**qPCR**
*toxR/rpoD*	10^2^	10^2^	10^3^	10^2^
*tdh*	10^2^	10^2^	10^4^	10^1^
*trh1*	10^3^	10^3^	10^4^	10^2^
*trh2*	10^3^	10^3^	10^4^	10^2^

The LAMP-XO assay exhibited comparable DLs to standard LAMP for all 4 sets of primers. Compared to conventional PCR, its DLs were 10 times more sensitive for *toxR*, *trh1*, and *trh2* and 100 folds greater for *tdh*. LAMP-XO also had equivalent DL to qPCR for *rpoD* but 10 times less sensitive for *tdh*, *trh1*, and *trh2*. This finding demonstrated that the LAMP-XO was as sensitive as standard LAMP and qPCR, but much more sensitive than PCR.

### Comparative detection limit of LAMP-XO, standard LAMP, conventional PCR, and qPCR, assays using spiked shrimp samples

**Figure 2:**
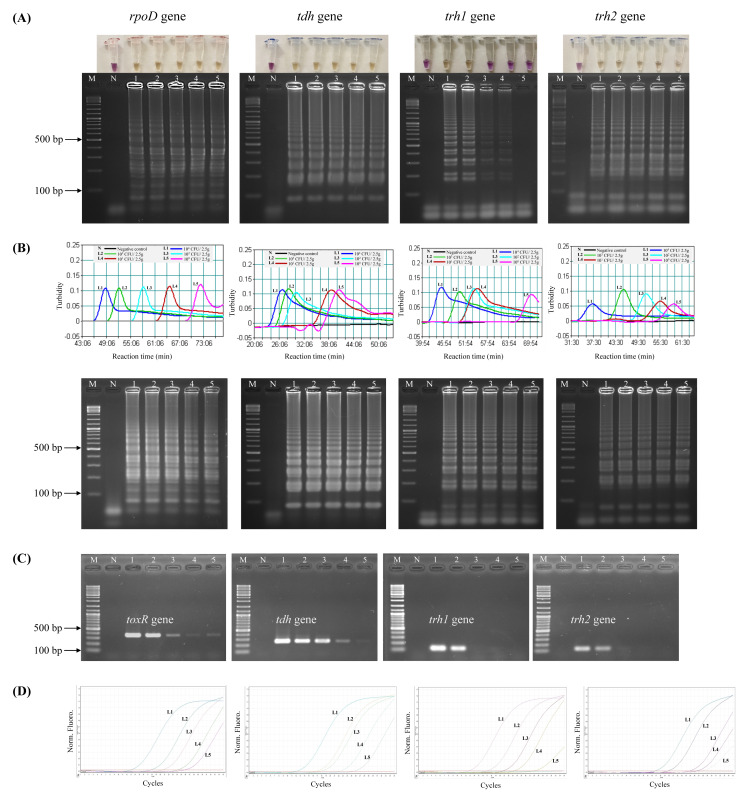
Comparative sensitivity of LAMP-XO, standard LAMP, conventional PCR, and qPCR assays for the detection of total *V. parahaemolyticus* and pathogenic *V. parahaemolyticus* using 10^4^–10^0^ CFU/2.5 g spiked shrimp. The *rpoD* primer was used for total *V. parahaemolyticus* detection in LAMP-XO, standard LAMP, and qPCR assays while the *toxR* primer was used in PCR assay. The *tdh*, *trh1*, and *trh2* primers were used for pathogenic *V. parahaemolyticus* detection in all assays. (A) LAMP-XO assay detected by the naked eye (top) and AGE (bottom); (B) Standard LAMP assay detected by a turbidimeter (top) and AGE (bottom); (C) Conventional PCR detected by AGE; (D) Quantitative PCR (q-PCR) assay. L1: 10^4^ CFU/2.5 g spiked shrimp, L2: 10^3^ CFU/2.5 g spiked shrimp, L3: 10^2^ CFU/2.5 g spiked shrimp, L4: 10^1^ CFU/2.5 g spiked shrimp, L5: 10^0^ CFU/2.5 g spiked shrimp, M: 1 kb DNA ladder, N: Negative control.

Prior to testing the analytical sensitivity of the assays, *V. parahaemolyticus*-negative shrimp samples were separately spiked with the reference strains at the concentrations of 10^4^–10^0^ CFU/2.5 g spiked shrimp. Amplification of *V. parahaemolyticus* gDNA was not observed by qPCR in a negative control (a non-spiked sample). The DL of LAMP-XO for *rpoD*, *tdh*, and *trh2* was 10^0^ CFU/2.5 g spiked shrimp, and for *trh1* primers was 10^3^ CFU/2.5 g spiked shrimp (Table 4, Fig. 2A, top). All of the colorimetric results were in accordance with the AGE results except for *trh1* detection where AGE could detect down to 10^2^ and 10^1^ CFU/2.5 g spiked shrimp (Fig. 2A, bottom). However, no distinct positive (yellow) test results were observed at these dilutions. The detection limits (DLs) of standard LAMP (Fig. 2B) and qPCR assays (Fig. 2D) for all 4 sets of primers were 10^0^ CFU/2.5 g spiked shrimp whereas those of PCR were 10^0^ and 10^1^ CFU/2.5 g spiked shrimp for *toxR* and *tdh*, respectively and 10^3^ CFU/2.5 g spiked shrimp for *trh1* and *trh2* (Fig. 2C).

**Table 4:** Comparative detection limit of LAMP-XO, standard LAMP, conventional PCR, and qPCR assays using a 10-fold serial dilution of spiked shrimp samples (10^4^–10^0^ CFU/mL).

**Primer**	**10-fold serial dilution of spiked samples (10** ^**4**^ **–** **10** ^**0**^ **CFU/mL)**			
	**LAMP-XO**	**Standard LAMP**	**Conventional PCR**	**qPCR**
*toxR/rpoD*	10^0^	10^0^	10^0^	10^0^
*tdh*	10^0^	10^0^	10^1^	10^0^
*trh1*	10^3^	10^0^	10^3^	10^0^
*trh2*	10^0^	10^0^	10^3^	10^0^

LAMP-XO assay revealed comparable DLs to standard LAMP and qPCR for *rpoD*, *tdh*, and *trh2* and 1,000 times less sensitive than that of qPCR for *trh1*. Compared to conventional PCR, limits of LAMP-XO detection were 10 times more sensitive for *tdh* and 1,000 times more sensitive for *trh2*. These findings were in accordance with those using a 10-fold serial dilution gDNA which demonstrated overall LAMP-XO was more sensitive than PCR whereas LAMP-XO sensitivity was comparable to those of standard LAMP and qPCR except for *trh1*.

### LAMP-XO assay for the detection of total *V. parahaemolyticus* (pathogenic and non-pathogenic strains) and pathogenic *V. parahaemolyticus* (*tdh*^+^ and/or *trh1*^+^ and/or *trh2*^+^) in raw seafood samples

LAMP-XO assay efficacy was validated using 102 raw seafood samples purchased from fresh markets and supermarkets and collected from shrimp farms in Thailand and North Vietnam. The colorimetric results were compared with the results from PCR and qPCR. Using qPCR as a gold standard, LAMP-XO and PCR assays identified 76/102 and 74/102 samples positive for *V. parahaemolyticus* contamination, respectively (Table 5). Thus, the clinical sensitivity, specificity, PPV, NPV, diagnostic accuracy, and percent overall agreement (POA) were 100% for all categories for LAMP-XO, but were 97.4%, 100%, 100%, 96.4%, 98.4%, and 98.0%, respectively, for PCR. No statistically significant difference was observed for *V. parahaemolyticus* detection between qPCR and LAMP-XO and between qPCR and PCR (*P* > 0.05).

**Table 5:** Comparison of qPCR, LAMP-XO, and conventional PCR for *V. parahaemolyticus* detection (*n* = 102 seafood samples).

	**No. of results for** **qPCR**							
**Method** **and result**	**Positive**	**Negative**	**Sensitivity, %** **(95% CI)**	**Specificity, %** **(95% CI)**	**PPV, %** **(95% CI)**	**NPV, %** **(95% CI)**	**Diagnostic** **accuracy (%)**	**POA (%)**
**qPCR**								
Positive	76	0						
Negative	0	26	100 (95.3–100)	100 (86.8–100)	100	100	100	100
**LAMP-XO**								
Positive	76	0						
Negative	0	26	100 (95.3–100)	100 (86.8–100)	100	100	100	100
**PCR**								
Positive	74	0						
Negative	2	26	97.4 (90.8–99.7)	100 (86.8–100)	100	96.4 (87.0–99.0)	98.4	98.0

For pathogenic strain detection, 76 samples positive for *V. parahaemolyticus* by a gold standard qPCR were tested for *tdh*, *trh1*, and *trh2* genes. LAMP-XO for *tdh* detection had sensitivity, specificity, PPV, and NPV of 90.5%, 100%, 100%, and 88%, respectively. The diagnostic accuracy and POA between LAMP and qPCR results were 94.4% and 97.4%, respectively (Table 6). PCR for *tdh* diagnosis showed 38.1% sensitivity, 98.2 specificity, 96.8% PPV, and 52.4% NPV. The diagnostic accuracy and POA between PCR and qPCR results were 62.7% and 81.6%, respectively (Table 6). No statistically significant difference was observed for *tdh* detection between qPCR and LAMP-XO (*P* > 0.05) while there were statistically significant differences between qPCR and PCR and between LAMP-XO and PCR (*P* < 0.05).

**Table 6:** Comparison of qPCR, LAMP-XO, and conventional PCR for *tdh* detection in 76 *V. parahaemolyticus*-positive samples.

	**No. of results for** **qPCR**							
**Method** **and result**	**Positive**	**Negative**	**Sensitivity, %** **(95% CI)**	**Specificity, %** **(95% CI)**	**PPV, %** **(95% CI)**	**NPV, %** **(95% CI)**	**Diagnostic** **accuracy (%)**	**POA (%)**
**qPCR**								
Positive	21	0						
Negative	0	55	100 (83.9–100)	100 (93.5–100)	100	100	100	100
**LAMP-XO**								
Positive	19	0						
Negative	2	55	90.5 (69.6–98.8)	100 (93.5–100)	100	88 (66.1–96.5)	94.4	97.4
**PCR** ^a^								
Positive	8	1						
Negative	13	54	38.1 (18.1–61.6)	98.2 (90.3–100)	96.8 (80–99.7)	52.4 (44–60.7)	62.7	81.6

**Notes.**

a There was a statistically significant difference for *tdh* detection between qPCR and PCR and between LAMP-XO and PCR (*P* < 0.05).

LAMP-XO for *trh1* detection demonstrated 75% sensitivity, 100% specificity, 100% PPV, and 73.5% NPV. The diagnostic accuracy, and POA between LAMP-XO and qPCR results were 85.3% and 96.1%, respectively (Table 7). PCR for *trh1* detection exhibited sensitivity, specificity, PPV, and NPV of 58.3%, 100%, 100%, and 62.5%, respectively. The diagnostic accuracy and POA between PCR and qPCR results were 75.4% and 93.4%, respectively (Table 7). No statistically significant difference was observed for *trh1* detection between qPCR and LAMP-XO and between qPCR and PCR (*P* > 0.05).

**Table 7:** Comparison of qPCR, LAMP-XO, and conventional PCR for *trh1* detection in 76 *V. parahaemolyticus*-positive samples.

	**No. of results for** **qPCR**							
**Method** **and result**	**Positive**	**Negative**	**Sensitivity, %** **(95% CI)**	**Specificity, %** **(95% CI)**	**PPV, %** **(95% C1I)**	**NPV, %** **(95% CI)**	**Diagnostic** **accuracy (%)**	**POA (%)**
**qPCR**								
Positive	12	0						
Negative	0	64	100 (73.5–100)	100 (94.4–100)	100	100	100	100
**LAMP-XO**								
Positive	9	0						
Negative	3	64	75 (42.8–94.5)	100 (94.4–100)	100	73.5 (51.1–88.1)	85.3	96.1
**PCR**								
Positive	7	0						
Negative	5	64	58.3 (27.7–84.8)	100 (94.4–100)	100	62.5 (46.1–76.5)	75.4	93.4

LAMP-XO for *trh2* detection exhibited 100% for sensitivity, specificity, PPV, NPV, diagnostic accuracy, and POA which were similar to those of qPCR. For *trh2* detection, PCR revealed 55.6% sensitivity, 100% specificity, 100% PPV, and 61% NPV. The diagnostic accuracy and POA between PCR and qPCR results were 73.8% and 89.5%, respectively (Table 8). The statistically significant difference was observed for *trh2* detection between qPCR and PCR and between LAMP-XO and PCR (*P* < 0.05).

**Table 8:** Comparison of qPCR, LAMP-XO, and conventional PCR for *trh2* detection in 76 *V. parahaemolyticus*-positive samples.

	**No. of results for** **qPCR**							
**Method** **and result**	**Positive**	**Negative**	**Sensitivity, %** **(95% CI)**	**Specificity, %** **(95% CI)**	**PPV, %** **(95% CI)**	**NPV, %** **(95% CI)**	**Diagnostic** **accuracy (%)**	**POA (%)**
**qPCR**								
Positive	18	0						
Negative	0	58	100 (81.5–100)	100 (93.8–100)	100	100	100	100
**LAMP-XO**								
Positive	18	0						
Negative	0	58	100 (81.5–100)	100 (93.8–100)	100	100	100	100
**PCR** ^a^								
Positive	10	0						
Negative	8	58	55.6 (30.8–78.5)	100 (93.3–100)	100	61 (48.3–72.4)	73.8	89.5

**Notes.**

a There was a statistically significant difference for *trh2* detection between qPCR and PCR and between LAMP-XO and PCR (*P* < 0.05).

Overall, LAMP-XO yielded results comparable to those of qPCR for *rpoD*, *tdh*, *trh1*, and *trh2* detection since no statistically significant difference was observed between the 2 methods. Compared to PCR, LAMP-XO significantly demonstrated greater performance for *tdh* and *trh2*. For *trh1*, LAMP-XO was prone to have higher performance to PCR. However, no statistically significant difference was observed for *trh1* detection between the 2 methods.

### Distribution of pathogenic genes in *V. parahaemolyticus*-positive samples

Based on the results of a gold standard qPCR and the presence or absence of the *tdh* or *trh1* or *trh2* toxin genes, the 76 *V. parahaemolyticus*-positive samples could be classified into 8 groups: *tdh*^+^/*trh1*^−^/*trh2*^−^, *tdh*^+^/*trh1*^+^/*trh2*^−^, *tdh*^+^/*trh1*^−^/*trh2*^+^, *tdh*^+^/*trh1*^+^/*trh2*^+^, *tdh*^−^/*trh1*^+^/*trh2*^−^, *tdh*^−^/*trh1*^+^/*trh2*^+^, *tdh*^−^/*trh1*^−^/*trh2*^+^, and *tdh*^−^/*trh1*^−^/*trh2*^−^ (Table 9). Most of *V. parahaemolyticus* isolates were *tdh*^−^/*trh1*^−^/*trh2*^−^ (64.5%, 49/76) and the rest were pathogenic strains with *tdh*^+^ and/or *trh1*^+^ and/or *trh2*^+^. Among pathogenic strains (27 isolates), *tdh*^+^/*trh1*^−^/*trh2*^+^ strains were predominant (11 isolates) followed by *tdh*^+^/*trh1*^+^/*trh2*^−^ (6 isolates), *tdh*^+^/*trh1*^+^/*trh2*^+^ (3 isolates), *tdh*^−^/*trh1*^−^/*trh2*^+^(3 isolates), *tdh*^−^/*trh1*^+^/*trh2*^−^ (2 isolates), *tdh*^+^/*trh1*^−^/*trh2*^−^ (1 isolate), and *tdh*^−^/*trh1*^+^/*trh2*^+^ (1 isolate).

**Table 9:** Characteristics of 76 *V. parahaemolyticus* isolates.

**Genotype**	**No. of isolates (%)**
*tdh* ^+^ */trh1* ^−^ */trh2* ^−^	1 (1.3%)
*tdh* ^+^ */trh1* ^+^ */trh2* ^−^	6 (7.9%)
*tdh* ^+^ */trh1* ^−^ */trh2* ^+^	11 (14.5%)
*tdh* ^+^ */trh1* ^+^ */trh2* ^+^	3 (3.95%)
*tdh* ^−^ */trh1* ^+^ */trh2* ^−^	2 (2.6%)
*tdh* ^−^ */trh1* ^+^ */trh2* ^+^	1 (1.3%)
*tdh* ^−^ */trh1* ^−^ */trh2* ^+^	3 (3.95%)
*tdh* ^−^ */trh1* ^−^ */trh2* ^−^	49 (64.5%)

## Discussion

Hygiene problems with seafood from cross-contamination in the seafood harvesting period from farm to fork lead to the increase of *V. parahaemolyticus* in the food chain (Hara-Kudo & Kumagai, 2014*).* To assure the safe seafood supply and to prevent economic losses, early monitoring and surveillance of *V. parahaemolyticus* are of utmost importance. In this study, a colorimetric assay based on LAMP-XO (Jaroenram, Cecere & Pompa, 2019) was developed, evaluated, and validated as an effective molecular tool to detect total and pathogenic *V. parahaemolyticus* in seafood. The factors, including the concentrations of dNTP mix, betaine, MgSO_4_, *Bst* 2.0 WarmStart DNA polymerase, and XO as well as reaction temperature and reaction time were optimized to obtain maximal amplification for each target gene. To accelerate the reaction amplification, we attempted to design the extra primers called multiple hybrid, inner primers (MHP) for *rpoD*, *tdh*, *trh1*, and *trh2* target genes. However, only MHP for *rpoD* were accomplished. The novel molecular method called “MHP-LAMP” developed by our group could be used successfully to increase the sensitivity and speed up the *rpoD* gDNA amplification (Lamalee et al., 2023). The MHP for the other 3 target genes could not be designed due to the limitation of the sequence length and sequence properties of the core primers.

Using a 10-fold serial dilution of gDNA, the DL of LAMP-XO for *rpoD* and *tdh* detection was 10^2^ copies/reaction and for *trh1* and *trh2*, it was 10^3^ copies/reaction. In sterilized Pacific white shrimp spiked with known quantities of the reference strains, LAMP-XO detected 10^0^ CFU/2.5 g spiked shrimp for *rpoD*, *tdh*, and *trh2* and 10^3^ CFU/2.5 g spiked shrimp for *trh1* within 4 h of pre-enrichment. Although no distinct positive (yellow) test results of LAMP-XO were observed for *trh1* at 10^2^ and 10^1^ CFU/2.5 g spiked shrimp, LAMP-XO products were observed at these dilutions by AGE. The discrepancy between LAMP-XO and AGE results was due to the fact that XO in LAMP reaction could not shift from purple to yellow because pH of LAMP reaction was > 6.7 due to the low amount of LAMP reaction by-products. These imply that the threshold of LAMP amplicons to trigger a distinctive purple-to-yellow readout is higher than that to allow a clearly visible result on AGE. Extending an incubation time to more than 90 min may help by promoting a color result development. Unlike *rpoD, tdh*, and *trh2* primers, the *trh1* primers lack of the LB primer to accelerate the reaction amplification resulting the decrease of the sensitivity of the *trh1*-LAMP-XO assay. When compared to conventional PCR, standard LAMP, and qPCR as a gold standard method, our LAMP-XO yielded results comparable to standard LAMP and qPCR except for *trh1* in spiked samples. However, the DL of LAMP-XO for *trh1* in spiked samples could be improved by increasing the reaction time to 90 min to increase LAMP reaction amplification. Compared to conventional PCR, LAMP-XO had greater sensitivity for *tdh* and *trh2* detection.

For the detection of *V. parahaemolyticus* using a 10-fold serial dilution of gDNA, our LAMP-XO demonstrated similar sensitivity to the study by Hu et al. (2021) (1.127 × 10^2^ copies/reaction) and demonstrated higher sensitivity than that of the study by Liu et al. (2017) (1.789 × 10^3^ copies/reaction). For the detection of *tdh* using a 10-fold serial dilution of gDNA, the LAMP-XO result had comparable sensitivity to that of the study by Anupama et al. (2021) (1.82 × 10^2^ copies/reaction). In spiked samples, the LAMP-XO result for *V. parahaemolyticus* detection showed higher sensitivity than those of the previous studies by Di et al. (2015) (2 CFU/g of 3-h spiked sample) and Zeng et al. (2014) (1.9 CFU/g of 6-h spiked sample).

To confirm the clinical sensitivity, specificity, and accuracy of LAMP-XO assay, 102 raw seafood samples were used. The results revealed that the performance of LAMP-XO assay for *V. parahaemolyticus rpoD, tdh*, *trh1*, and *trh2* detection was comparable to a gold standard qPCR and significantly superior to conventional PCR for *tdh* and *trh2* detection as analyzed by a McNemar chi-square test. These results indicate that the LAMP-XO assay developed in this present study is a better choice than PCR and qPCR for routine detection of *V. parahaemolyticus* in naturally contaminated seafood samples and in environment since this method does not require expensive equipment and well-trained personnel and has a short turnaround time.

The occurrence of *tdh* and/or *trh* in environmental *V. parahaemolyticus* isolates is normally 1–10% depending on locations, sample sources, and detection methods (Raghunath, 2015). The LAMP-XO results showed that 35.5% (27/76) of *V. parahaemolyticus* detected in this present study were pathogenic strains (*tdh*^+^ and/or *trh1*^+^ and/or *trh2*^+^). The *tdh*^+^/*trh1*^−^/*trh2*^+^ strain was the most frequently observed (11/76;14.5%). In this finding, the coexistence of *trh1* and *trh2* were observed in *tdh*^+^/*trh1* ^+^/*trh2*^+^ (3/76; 3.95%) and *tdh*^−^/*trh1*^+^/*trh2*^+^ (1/76; 1.3%) strains. The *tdh*^+^/*trh1*^+^/*trh2*^+^ and *tdh*^−^/*trh1*^+^/*trh2*^+^ strains were obtained from fresh market and supermarket samples, respectively. However, previous studies demonstrated that *V. parahaemolyticus* isolates carrying *trh2* did not contain *trh1* (Kishishita et al., 1992; Kongrueng et al., 2018). The discrepancy of the results is probably due to mixed populations of *V. parahaemolyticus* carrying *trh1* and *V. parahaemolyticus* carrying *trh2* in the same samples since the detection of *rpoD*, *tdh*, *trh1*, and *trh2* in these 4 samples was done directly from the seafood samples after enrichment. In this present study, the number of *trh*^+^ *V. parahaemolyticus* was prone to be higher than that of *tdh*^+^ *V. parahaemolyticus.*

It is worth noting that although we have reported the LAMP primer set (Lamalee et al., 2023), this does not lower the value of the present study. This is because our previous publication focuses on introducing the new concept of using additional hybrid LAMP primers to enhance the diagnostic sensitivity of a typical LAMP assay, and validate it at a proof-of-concept level by using *rpoD*-LAMP for *V. parahaemolyticus* detection as a fundamental model. However, in our present study, we extended our finding by transforming it into a colorimetric, xylenol orange (XO)-based LAMP assay with the naked-eye readout format to enable simplicity yet having detection efficiency as a more complicated PCR-based protocol. To the best of our knowledge, LAMP-XO has not been applied to detect *V. parahaemolyticus*. This brings about the novelty of this study to some extent. In addition, the significance of this study is that the LAMP-XO assay showed 100% reliability in detecting both pathogenic and non-pathogenic *V. parahaemolyticus* (by *rpoD* and *trh2* genes) as well as discriminating them (by *trh2* gene). Thus, they could bridge the gap by complementing or replacing the current diagnostic methods as a quick and reliable assay while confirmatory *V. parahaemolyticus* diagnosis are processed by slower and more expensive conventional methods such as real-time PCR.

## Conclusions

Global outbreaks caused by pathogenic *V. parahaemolyticus* are recurrent, emphasizing the requirement for effective control of contaminants in seafood. The LAMP-XO assay reported here is rapid, simple, practical, cost-effective, and as efficient as qPCR. Thus, this assay is suitable to facilitate surveillance for total *V. parahaemolyticus* (pathogenic and non-pathogenic strains) and pathogenic *V. parahaemolyticus* (*tdh*^+^ and/or *trh1*^+^ and/or *trh2*^+^) contamination in seafood, screening of contaminated seafood prior to consumption, and examinations to detect the food poisoning causative agents. This assay is also useful for ecological research related to environmental factors, seasons, areas, and practices. From our experience, this assay can be further improved to make it more efficient for the weak positive reaction, for example, by increasing the amplification time for *trh1* from 75 min to 90 min and increasing the XO concentration for *trh2* from 0.03 mM to 0.06 mM. The use of a one-step LAMP-XO colorimetric assay together with the addition of the MHP based on the previously reported core primer set are the concepts that can be applied to boost sensitivity and rapidity of other existing LAMP-based assays. It could aid in reducing the cost and time in redesigning a whole new primer set from the beginning.

## Supplemental Information

10.7717/peerj.16422/supp-1 Supplemental Information 1 Reagent concentrations of the initial standard protocol for LAMP-XO optimization

10.7717/peerj.16422/supp-2 Supplemental Information 2 Design of parameter optimization in LAMP-XO assay

10.7717/peerj.16422/supp-3 Supplemental Information 3 PCR primers and conditions used in this study

10.7717/peerj.16422/supp-4 Supplemental Information 4 QPCR primers and conditions used in this study

10.7717/peerj.16422/supp-5 Supplemental Information 5 Comparison of the results of 102 raw seafood samples among LAMP-XO, conventional PCR, and qPCR assays

10.7717/peerj.16422/supp-6 Supplemental Information 6 Comparison of LAMP-XO, conventional PCR and qPCR assays

10.7717/peerj.16422/supp-7 Supplemental Information 7 Detection of total *V. parahaemolyticus* (pathogenic and non-pathogenic strains) and pathogenic *V. parahaemolyticus* (*tdh*^+^ and/or *trh1*^+^ and/or *trh2*^+^) in 102 raw seafood samples using LAMP-XO, PCR, a

## Additional Information and Declarations

### Data Availability

The following information was supplied regarding data availability: The raw data are available in the Supplementary File.
