# Supplementary material for: End-point rapid detection of total and pathogenic Vibrio parahaemolyticus (tdh+ and/or trh1+ and/or trh2+) in raw seafood using a colorimetric loop-mediated isothermal amplification-xylenol orange technique
Source: PeerJ. 2024 Jan 3;12:e16422. doi: 10.7717/peerj.16422 (PMC10771086; doi:10.7717/peerj.16422)
Supplement: Supplemental Information 1 [file peerj-12-16422-s001.docx]

**Table S1** Reagent concentrations of the initial standard protocol for LAMP-XO optimization

| **Component** | **Final Concentration** |
| --- | --- |
| 10× low buffer (pH 8.5) | 1× |
| dNTP Mix (10 mM) | 1.4 mM |
| MgSO_4_ (100 mM) | 6 mM |
| Betaine (5M) | 0.4 M |
| *Bst* 2.0 WarmStart DNA polymerase (8,000 U/ml) | 8 U |
