# Supplementary material for: End-point rapid detection of total and pathogenic Vibrio parahaemolyticus (tdh+ and/or trh1+ and/or trh2+) in raw seafood using a colorimetric loop-mediated isothermal amplification-xylenol orange technique
Source: PeerJ. 2024 Jan 3;12:e16422. doi: 10.7717/peerj.16422 (PMC10771086; doi:10.7717/peerj.16422)
Supplement: Supplemental Information 2 [file peerj-12-16422-s002.docx]

| **Reaction condition (unit)** | **Parameter with increasing order** |
| --- | --- |
| Reaction temperature (°C) | 60, 63, 65 |
| dNTP Mix (mM) | 1.2, 1.4, 1.6, 1.8 |
| MgSO_4_ (mM) | 4, 6, 8,10 |
| Betaine (M) | 0.2, 0.4, 0.6, 0.8 |
| *Bst* 2.0 WarmStart DNA polymerase (U) | 6, 8, 10, 12 |
| Reaction time (min) | 30, 45, 60, 75, 90 |
| Xylenol orange (mM) | 0.03, 0.06, 0.09, 0.12 |

**Table S2** Design of parameter optimization in LAMP-XO assay
