# Supplementary material for: End-point rapid detection of total and pathogenic Vibrio parahaemolyticus (tdh+ and/or trh1+ and/or trh2+) in raw seafood using a colorimetric loop-mediated isothermal amplification-xylenol orange technique
Source: PeerJ. 2024 Jan 3;12:e16422. doi: 10.7717/peerj.16422 (PMC10771086; doi:10.7717/peerj.16422)
Supplement: Supplemental Information 3 [file peerj-12-16422-s003.docx]

**Table S3** PCR primers and conditions used in this study

| **Primer name** | **Sequence (5′ to 3′)** | **Target gene** | **Amplicon size (bp)** | **Reference** | **PCR Condition** |
| --- | --- | --- | --- | --- | --- |
| *toxR*-F | GTCTTCTGACGCAATCGTTG | *toxR* | 368 | *Kim et al., 1999* | 94 °C-1 min; 63 °C-1.30 min; 72 °C-1.30 min |
| *toxR*-B | ATACGAGTGGTTGCTGTCATG |  |  |  |  |
|  |  |  |  |  |  |
| *tdh*-F | CCACTACCACTCTCATATGC | *tdh* | 251 | *Tada et al., 1992* | 95 °C-1 min; 58 °C-1 min; 72 °C-1 min |
| *tdh*-B | GGTACTAAATGGCTGACATC |  |  |  |  |
|  |  |  |  |  |  |
| *trh1*-F | AAAAGCGTTCACGGTCAATC | *trh1* | 110 | *Messelhäusser et al., 2010* | 94 °C-1 min; 56 °C-1 min; 72 °C-1.30 min |
| *trh1*-B | CCAGAAAGAGCAGCCATTGT |  |  |  |  |
|  |  |  |  |  |  |
| *trh2*-F | CCCCAGTTAAGGCAATTGTG | *trh2* | 120 | *Messelhäusser et al., 2010* | 95 °C-1 min; 59.5 °C-1 min; 72 °C-1 min |
| *trh2*-B | AGGCGCTTAACCACTTTGAA |  |  |  |  |
