# Supplementary material for: End-point rapid detection of total and pathogenic Vibrio parahaemolyticus (tdh+ and/or trh1+ and/or trh2+) in raw seafood using a colorimetric loop-mediated isothermal amplification-xylenol orange technique
Source: PeerJ. 2024 Jan 3;12:e16422. doi: 10.7717/peerj.16422 (PMC10771086; doi:10.7717/peerj.16422)
Supplement: Supplemental Information 4 [file peerj-12-16422-s004.docx]

**Table S4** qPCR primers and conditions used in this study

| **Primer name** | **Sequence (5′ to 3′)** | **Target gene** | **Amplicon size (bp)** | **Reference** | **qPCR Condition** |
| --- | --- | --- | --- | --- | --- |
| *rpoD*-F3 | ACCAGCTACGCAGCACA | *rpoD* | 272 | *Nemoto et al., 2011* | 95 °C-20 s; 55 °C-34 s; 72 °C-45 s |
| *rpoD*-B3 | CACTTGATTCGTTACCAGTGAATAGG |  |  |  |  |
|  |  |  |  |  |  |
| *tdh*-F3 | GTCTCTGACTTTTGGACAAACCG | *tdh* | 310 | *Nemoto et al., 2009* | 95 °C-20 s; 55 °C-34 s; 72 °C-45 s |
| *tdh*-B3 | CTACATTAACAAAATATTCTGGAGTTTCATCC |  |  |  |  |
|  |  |  |  |  |  |
| *trh1*-F3 | GCGCCTATATGACGGTAA | *trh1* | 211 | *Yamazaki et al., 2010* | 95 °C-20 s; 55 °C-34 s; 72 °C-45 s |
| *trh1*-B3 | ACATTGACGAAATATTCTGGC |  |  |  |  |
|  |  |  |  |  |  |
| *trh2*-F | CCCCAGTTAAGGCAATTGTG | *trh2* | 120 | *Messelhäusser et al., 2010* | 95 °C-20 s; 55 °C-34 s; 72 °C-45 s |
| *trh2*-B | AGGCGCTTAACCACTTTGAA |  |  |  |  |
