# Supplementary material for: End-point rapid detection of total and pathogenic Vibrio parahaemolyticus (tdh+ and/or trh1+ and/or trh2+) in raw seafood using a colorimetric loop-mediated isothermal amplification-xylenol orange technique
Source: PeerJ. 2024 Jan 3;12:e16422. doi: 10.7717/peerj.16422 (PMC10771086; doi:10.7717/peerj.16422)
Supplement: Supplemental Information 5 [file peerj-12-16422-s005.docx]

**Table S5** LAMP-XO optimization for *rpoD*, *tdh*, *trh1*, and *trh2* detection with various concentrations of XO at 0.03, 0.06, 0.09, and 0.12 mM using 10^6^ copies/mL of reference strain gDNA.
