# Supplementary material for: End-point rapid detection of total and pathogenic Vibrio parahaemolyticus (tdh+ and/or trh1+ and/or trh2+) in raw seafood using a colorimetric loop-mediated isothermal amplification-xylenol orange technique
Source: PeerJ. 2024 Jan 3;12:e16422. doi: 10.7717/peerj.16422 (PMC10771086; doi:10.7717/peerj.16422)
Supplement: Supplemental Information 6 [file peerj-12-16422-s006.docx]

**Table S6** Comparison of LAMP-XO, conventional PCR, and qPCR assays

|  | **LAMP-XO** | **PCR** | **qPCR** |
| --- | --- | --- | --- |
| Temperature cycle | Isothermal amplification (60–65 °C) | Different temperature cycles required | Different temperature cycles required |
| Equipment | Heat block | Thermocycler | Thermocycler |
| Product detection | Naked eye | Electrophoresis | Real-time protocol |
| Analysis time | 75–90 min | 160 min | 130 min |
| Cost/1 reaction | ~ 0.54 US$ | ~ 0.79 US$ | ~ 0.76 US$ |
| Ease of use | Easy to carry out | Requires trained personnel | Requires trained personnel |
